# Origami-Inspired Vacuum-Actuated Foldable Actuator Enabled Biomimetic Worm-like Soft Crawling Robot

**DOI:** 10.3390/biomimetics9090541

**Published:** 2024-09-06

**Authors:** Qiping Xu, Kehang Zhang, Chenhang Ying, Huiyu Xie, Jinxin Chen, Shiju E

**Affiliations:** Key Laboratory of Urban Rail Transit Intelligent Operation and Maintenance Technology & Equipment of Zhejiang Province, Department of Robotics Engineering, College of Engineering, Zhejiang Normal University, Jinhua 321004, China; xuqiping@zjnu.edu.cn (Q.X.); yijidayu@163.com (K.Z.); hufei3277610753@163.com (C.Y.); 13789174734@163.com (H.X.)

**Keywords:** origami-inspired, vacuum-actuated, biomimetic worm-like, soft crawling robot, asymmetric structural design, multimodal locomotion, multifunctional characteristics

## Abstract

The development of a soft crawling robot (SCR) capable of quick folding and recovery has important application value in the field of biomimetic engineering. This article proposes an origami-inspired vacuum-actuated foldable soft crawling robot (OVFSCR), which is composed of entirely soft foldable mirrored origami actuators with a Kresling crease pattern, and possesses capabilities of realizing multimodal locomotion incorporating crawling, climbing, and turning movements. The OVFSCR is characterized by producing periodically foldable and restorable body deformation, and its asymmetric structural design of low front and high rear hexahedral feet creates a friction difference between the two feet and contact surface to enable unidirectional movement. Combining an actuation control sequence with an asymmetrical structural design, the body deformation and feet in contact with ground can be coordinated to realize quick continuous forward crawling locomotion. Furthermore, an efficient dynamic model is developed to characterize the OVFSCR’s motion capability. The robot demonstrates multifunctional characteristics, including crawling on a flat surface at an average speed of 11.9 mm/s, climbing a slope of 3°, carrying a certain payload, navigating inside straight and curved round tubes, removing obstacles, and traversing different media. It is revealed that the OVFSCR can imitate contractile deformation and crawling mode exhibited by soft biological worms. Our study contributes to paving avenues for practical applications in adaptive navigation, exploration, and inspection of soft robots in some uncharted territory.

## 1. Introduction

The locomotion mechanism observed in nature, particularly in mollusks, has served as a significant inspiration for fabricating soft robots with diverse motion capabilities. Soft robots capable of crawling [1], climbing [2], grasping [3], swimming [4], growing [5], rolling [6], and flying [7] have been developed by adopting different actuation mechanisms, which principally comprise fluidic pressure [8], chemical reaction [9], active materials [10], etc. Among these soft robots, the bioinspired soft crawling robots (SCRs) are increasingly gaining attention due to their more reliable interaction with humans or unpredictable surroundings, better adaptability, stronger robustness, and greater agility compared to traditional hard counterparts, which enables them to be competent for disaster search and rescue, drug delivery, pipeline inspection, intestinal diagnosis, military reconnaissance, and covert surveillance. Classic examples of SCRs include the quadruped-inspired type [11,12], earthworm-inspired type [13,14], inchworm-inspired type [15,16], caterpillar-inspired type [17,18], snake-inspired type [19,20], and cockroach-inspired type [21]. Nevertheless, origami-inspired biomimetic SCRs capable of folding and recovery are relatively less explored, and their foldability and restorability may play a crucial role in their body deformation and interaction with unknown environments.

Over the past few years, fluid-driven soft robots, particularly those powered by pneumatic actuation, have become more prevalent and mainstream than those driven by other actuation approaches [22,23], and are potential candidates in robotic application fields on account of their unique features such as continuously adjustable body deformation, simple actuation control, and harmonious environmental interactivity. Most prior SCRs constituted by a soft pneumatic actuator (SPA) are usually actuated by applying positive pressure. Typical cases include a pneumatically powered multigait quadruped SCR [24], a fully untethered kirigami-skinned SCR comprising an actuator covered with kirigami skins (moving like a snake) [25], a peristaltic SCR assembled with two radial SPAs and one axial SPA (creeping horizontally and vertically inside a cylinder) [26], a tethered winding-styled SCR made up of two winding SPAs and a telescopic SPA (turning around, climbing a vertical rod, carrying a payload) [27], a multimodal SCR covering three multi-bladder SPAs (linear movement, crossing, climbing) [28], and several modular SCRs constructed with an axial elongation SPA and a dual bending SPA (crawling and turning) [29]. However, these SCRs driven by positive-pressure actuation have some inherent shortfalls such as large radial expansion deformation, unreliable sealing, and abrupt bursting.

It is worth noting that negative pressure (i.e., vacuum) actuation is increasingly becoming a hopeful actuation technique and bringing some benefits [30]. One major benefit of vacuum-powered soft robots lies in decreasing rather than increasing volume, contraction rather than expansion in deformation (because the void spaces contract rather than expand upon actuation), and collapsing rather than bursting in chambers, which enables them to be applied in confined intestines or pipes. Relatively speaking, these robots have lower strain, longer fatigue life, better resilience, and stronger stability than positive-pressure driven robots. Furthermore, they can still work normally even if experiencing some small punctures as atmosphere pressure compresses chambers to enable them to be automatic sealing [31]. Consequently, exploring vacuum-actuated SCRs may bring about innovative solutions to some problems encountered in the field of soft robotics.

The recent development of vacuum-actuated actuators has inspired the creation of several new models of SCR. A late-model SCR (a tube-climbing robot) based on vacuum-actuated bucking actuators has the capability of climbing, cleaning, and navigating inside a tube under either desiccated or underwater circumstances [31]. A scalable, reconfigurable SCR integrated with vacuum-powered SPA modules enables multimodal locomotion containing a wave gait, rolling gait, and climbing gait [32]. A four-legged SCR combining four vacuum-actuated rotating actuators can mimic a reptilian walking gait in a diagonal coordination manner [33]. A rotatable and pipe-climbing SCR consists of several vacuum-powered twisting actuators having the power to fulfill turning, rotating, and climbing gaits [34]. A versatile SCR composed of two vacuum-driven spring actuators and two electrostatic actuators is able to realize two motion modes and obstacle navigation, climb a vertical wall, pass through a gap, and kick a ball [35]. A snake-like SCR made of soft silicone twisting actuators can execute rotational motion and pass through a pipe [36]. Nevertheless, the SCRs reported above are either complicated in structural design and programming control, are not completely soft (embedded with some hard parts or structures), or cannot easily realize quick contraction and restoration deformation. Therefore, it is necessary to develop some fully soft SCRs with a simple architecture, effortless fabrication process, reliable control strategy, and rapid deformation movement.

Taking inspiration from soft worms’ crawling locomotion mode, we developed a tethered origami-inspired vacuum-actuated foldable soft crawling robot (OVFSCR) that consists of origami actuators with a Kresling crease pattern and hexahedral feet. We designed (I) vacuum-driven mirrored origami actuators that generate periodically linear contractile deformation, (II) an asymmetric structure combined with a friction difference on two feet enabling unidirectional movement, and (III) a programmable control scheme that regulates the inflation and deflation interval to accomplish a crawling motion. Compared with some existing SCRs, our SCR exhibits superiority in terms of performance metrics including linear speed, turning speed, self-weight, payload/self-weight, response time, stability, and durability. The developed OVFSCR can fulfill multimodal locomotion and possess multifunctional characteristics.

## 2. Materials and Methods

### 2.1. Structural Design of the OVFSCR

**Figure 1:**
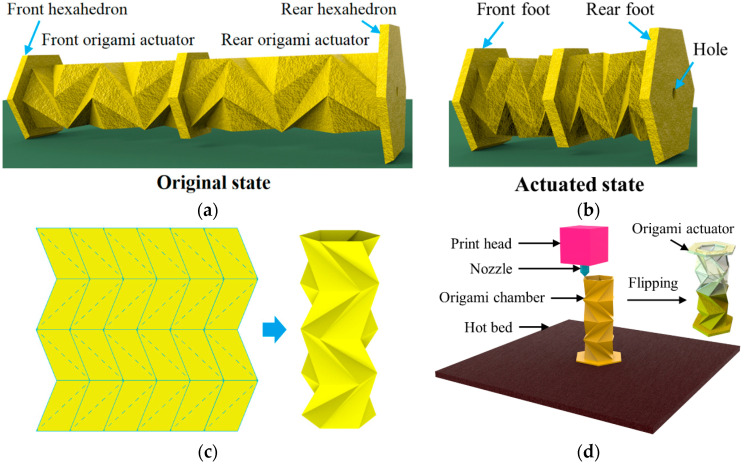
Schematic of an OVFSCR. (**a**) The robot consists of two origami actuators and two different hexahedral feet. Made from soft thermoplastic elastomer, the robot is in a still state when no vacuum pressure is applied. (**b**) The robot contracts linearly and is in actuated state when subjected to vacuum pressure. (**c**) An antisymmetric four-layer origami chamber; the blue dashed lines denote the Kresling crease pattern. (**d**) Three-dimensional printing fabrication process of an origami actuator.

Inspired by contractile locomotion of soft worms and folding deformation of origami structures, we designed an OVFSCR comprising mirrored origami actuators as the main body and hexahedrons as the feet, which is different from the SCR composed of arrayed twisting actuators [36]. The robot’s original state and actuated state subjected to vacuum pressure are shown in Figure 1a and Figure 1b, respectively. A single hollow origami actuator contains an antisymmetric four-layer origami chamber (layer height of 15 mm, wall thickness of 0.7 mm, relative angle of 60° for a single-layer origami chamber) with a Kresling crease pattern (Figure 1c). The front and rear origami actuators are securely bonded together with epoxy adhesive to form the robot’s main body. The front hexahedron (side length of 20 mm, thickness of 5 mm) is designed to be lower than the rear hexahedron (side length of 23 mm, thickness of 6 mm) with a central circular hole of 3.6 mm, and both hexahedrons have inclined bottom surfaces (a slope of 20° to the ground). The robot’s entire structure can be fully fabricated using soft thermoplastic elastomer (TPE) with high resilience in a 3D printer (Truth-2X), and the fabrication process is shown in Figure 1d. The finalized OVFSCR has a body length of 143 mm and a self-weight of 22.5 g.

The OVFSCR’s ingenuity lies in designing the rear foot’s inclination and integrating two origami actuators and two hexahedral feet into a single piece, which only needs a tethered tube to connect the robot with an external vacuum pump for realizing quick actuation. Upon applying a vacuum to the interconnected hollow origami chamber of the OVFSCR, the robot contracts along the longitudinal direction to generate axially linear contractile motion as the antisymmetric torsional Kresling origami structure counteracts twisting deformation and limits radial expansion deformation of the actuator (Figure 1b), which is a demanding challenge for most positive-pressure driven SCRs. In comparison to those SCRs powered by other actuation methods [22,23], the OVFSCR demonstrates smaller volume changes, higher efficiency, lower strain, and better stability, and it can rapidly recover to its original state once application of the vacuum pressure is ceased.

### 2.2. Crawling Locomotion Mechanism

One end of the soft tube is connected to the robot’s interior through a circular hole in the rear hexahedron, and the other end is connected to a vacuum pump (Fujiwara-1550D, −100~0 kPa) to actuate the OVFSCR. Once a vacuum is exerted, the robot will contract to an actuated state (Figure 1b), and the degree of contraction largely depends on the magnitude of the applied vacuum pressure, until it approaches a plateau at a vacuum pressure of 99.8 kPa. By actuating the OVFSCR in a specific control sequence, a fundamental periodic crawling gait is demonstrated. A peristaltic wave passing through the robot’s main body is generated, which enables the robot to achieve unidirectional continuous crawling locomotion.

**Figure 2:**
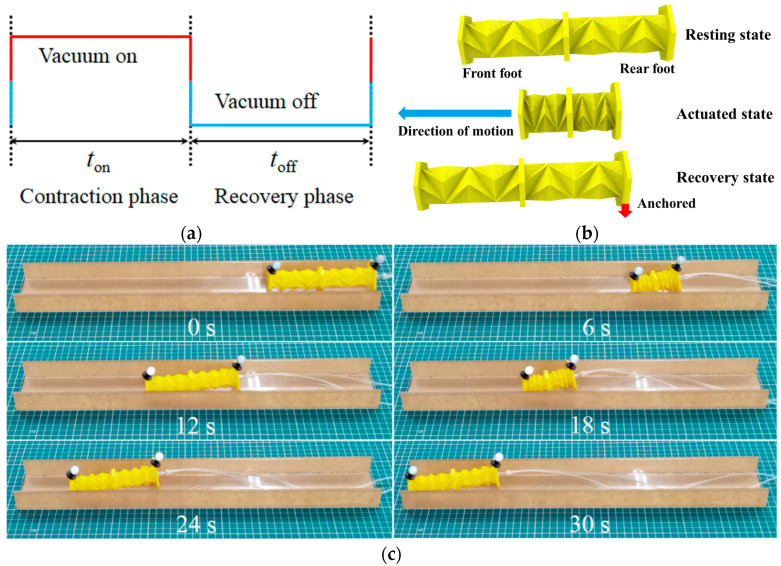
Locomotion schematic and crawling gait of the OVFSCR. (**a**) Vacuum actuation sequence of the robot for enabling crawling locomotion. The red line denotes the vacuum applied to the robot’s main body, whereas the blue line denotes no vacuum. (**b**) Forward crawling motion of the OVFSCR can be realized by following the periodic actuation sequence of three states. (**c**) A sequence of snapshots of the OVFSCR crawling on a dry smooth acrylic straight groove (length of 500 mm), and the robot carries with two fluorescent markers weighing 2.8 g via vacuum actuation in three sequential states. Crawling locomotion is controlled by tuning magnitude and duty cycle (i.e., two time constants, *t*_on_: time of vacuum on; *t*_off_: time of vacuum off) of vacuum pressure. The duration of the contraction phase and recovery phase can be varied to attain fast crawling motion.

As illustrated in Figure 2a, the locomotion gait of the OVFSCR involves a periodic actuation sequence of three states. First, starting with the state in which the robot’s two hexahedral feet are in line contact with the ground (resting state in Figure 2b). Second, the main body is actuated by applying vacuum, and the contractile force generated by the vacuum surpasses the frictional forces of the front and rear feet, which makes the robot contract towards the middle (actuated state in Figure 2b). Third, upon releasing the vacuum, the inclined surface of the rear foot tilts backwards, which enables the robot’s rear foot to be anchored to prevent backward slipping. The restoring force induced by the body restoration deformation propels the robot forward (recovery state in Figure 2b), and thus the robot moves forward. By cyclically controlling vacuum on and vacuum off with this actuation mode, the OVFSCR can mimic the peristaltic movement of biological worms.

By repeating the above actuation sequence, the OVFSCR can achieve continuous forward locomotion. The crawling motion of the robot can be controlled by actively tuning the magnitude and duty cycle of the vacuum pressure. Compared with prior SCRs, the obvious benefits of the proposed OVFSCR lies in quick folding and recovery, as well as fast locomotion speed. In addition, friction sheets with different friction coefficients can be attached to the front and rear feet to prevent backward slippage and minimize forward frictional resistance.

Benefiting from rapidly switchable contraction and recovery forces generated by vacuum pressure, the OVFSCR can locomote unidirectionally by controlling the simple actuation sequence. Figure 2c and Supplementary Movie demonstrate the unidirectional crawling locomotion after optimizing the duty cycle (i.e., the duration of each actuation sequence in Figure 2a). Measuring from the video through a 3D motion capture system (OptiTrack Prime 41), the robot crawls in the left direction, analogous to soft worms creeping on the ground, and attains an average speed of 11.9 mm/s (0.083 body length/s) using the optimal actuation sequence.

The OVFSCR is different from some previously proposed SCRs in that it has higher speed than that of the positive-pressure actuated SCR (0.053 body length/s) [12] and the SMA-actuated SCR (0.018 body length/s) [15]. Moreover, the robot can crawl on slippery flat surfaces, slopes, or tubes with variable diameters. It can also pass through circular tubes with a diameter greater than the circumscribed circle diameter of the rear hexahedron; otherwise, it may get stuck.

### 2.3. Theoretical Modeling of the OVFSCR

**Figure 3:**
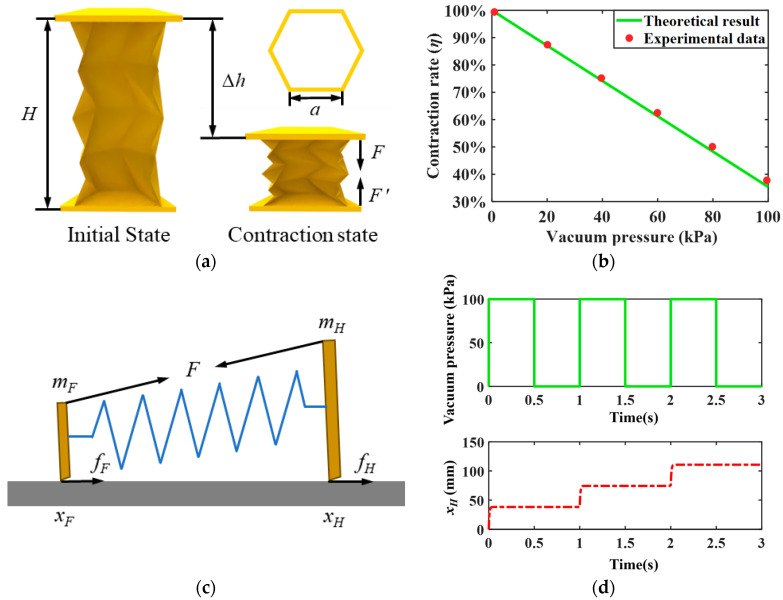
Schematics of the origami actuator and the theoretical model of the OVFSCR. (**a**) Deformation diagram of the actuator after applying vacuum pressure. (**b**) Contraction rate of the actuator versus vacuum pressure. (**c**) The body of the robot is characterized by one spring and two mass blocks, and an actuation force is denoted by *F*. Frictional forces *f_F_* and *f_H_* are exerted on the virtual front and rear feet. (**d**) Vacuum pressure versus time and current position *x_H_* of the rear foot versus time, and the robot locomotes intermittently.

Figure 3a exhibits the schematic of the single origami actuator with some geometric parameters: initial length *H*, deformation length Δ*h*, and internal side length *a*. Thus, we can obtain the internal hexagon’s cross-sectional area *S* of the origami actuator and the contraction force *F* caused by vacuum pressure *P*.

(1) $$S=3\sqrt{3}a^{2}/2$$

(2) F=PS

A static model is established to characterize the intrinsic relationship between vacuum pressure and compression deformation. The origami actuator is compressed by applying a vacuum, whereas its restoration lies on the resilience of the TPE material. The static model follows the fact that contraction force *F* balances elastic restoring force *F*′ in a stable state (Figure 3a). The restoring force is represented as:

(3) F′=k ∆h

where *k* is the elastic coefficient of the origami actuator, which relies on the inherent property of TPE material. To calculate *k*, it is assumed that the actuator is uniformly compressed. We then measured deformation length and contraction forces under different vacuum pressures along the axial direction by a dynamometer (HP-20; HANDPI), and *k* is equal to 1.096 N/mm after calculation. Thus, the relationship between deformation length Δ*h* and vacuum pressure *P* is formulated as:

(4) ∆h=PS/k

We can further define the contraction rate *η* to describe the above relationship:

(5) η=(H −∆h)/H

According to Equation (5), the solved theoretical result is shown in Figure 3b (denoted by the green solid line) and coincides with the experimental data (denoted by red circles). This demonstrates that contraction deformation of the actuator is linearly related to vacuum pressure; it increases with the rise in vacuum pressure and attains a maximum at a vacuum pressure of 100 kPa.

Moreover, a dynamic model is established to depict the robot’s motion features. If the actuation force is lower than the maximum static frictional force, the feet keep still; conversely, the feet will walk. In short, the robot is subjected to two types of dry friction (i.e., static friction and dynamic friction). Upon applying a vacuum, the robot contracts towards the middle, and the middle part can be regarded as a virtual front foot.

To simplify dynamic modeling, we ignore the motion of the real front foot, and only consider the motion of the virtual front foot and rear foot. In view of the OVFSCR’s locomotion gait, the virtual front and rear feet anchor and move alternately, and the robot can be conceptualized as a system consisting of one spring and two mass blocks (Figure 3c). The frictional forces on the virtual front foot *m_F_* and the rear foot *m_H_* are equivalent to *f_F_* = *f_H_* = 1/2*f*, and the total frictional force *f* = *μmg* can be experimentally measured using the dynamometer, where *μ* (about 0.02) signifies the dynamic friction coefficient that can be calculated through experimental testing [27]. The positions (*x_F_* and *x_H_*) of the virtual front and rear feet are coupled by the spring connected between two mass blocks, and the spring has an equivalent stiffness coefficient (i.e., elastic coefficient *k*) and an actuation force *F*. The spring force and actuation force are continuously updated during the calculation of the current position *x_F_* and *x_H_*. By uniformly dividing the robot’s total mass (22.5 g) into the virtual front foot (11.25 g) and the rear foot (11.25 g), we can model the robot’s inertial effect.

Starting from the resting state (Figure 2b), the actuation force induced by the vacuum pressure pulls the rear foot forward, the virtual front foot is considered to be anchored, and the equation of motion for the rear foot is:

(6) $$m_{H\;}d^{\;2}x_{H}/dt^{\;2}=F-k(l-x_{F}+x_{H})-f_{H}$$

When the rear foot stops, the restoring force pushes the virtual front foot forward, the rear foot is anchored, and the equation of motion for the virtual front foot is:

(7) $$m_{F\;}d^{\;2}x_{F}/dt^{\;2}=F+k(l-x_{F}+x_{H})-f_{F}$$

The above dynamic equations can be numerically solved by adopting the ODE45 function based on the explicit Runge–Kutta method. Figure 3d exhibits the relationship between vacuum pressure and displacement of the rear foot. On the whole, the rear foot follows a ladder-shaped displacement motion law, undergoes an abrupt locomotion upon applying vacuum pressure, and stays anchored after the vacuum is released. This motion behavior enables the robot to move forward like a one-way ratchet, which aligns well with the actual movement observed in the experiments.

## 3. Results

### 3.1. Crawling on Flat Surfaces with Different Substrates

**Figure 4:**
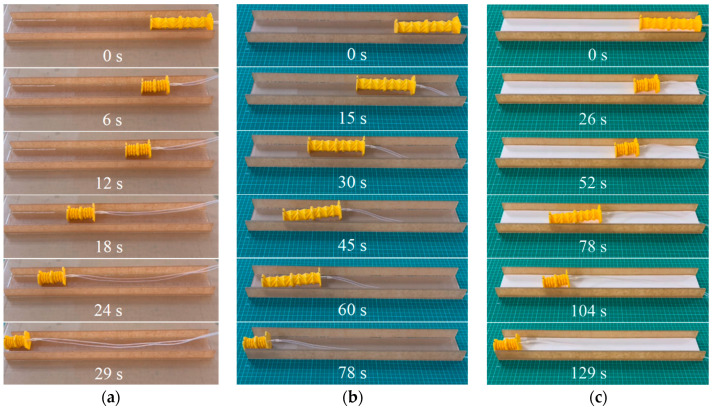
Demonstrations of the OVFSCR crawling horizontally on different substrate surfaces. The robot locomotes on (**a**) a slippery; (**b**) dry smooth; and (**c**) semi-rough acrylic plate groove surfaces. Different contact surfaces have various effects on the crawling speed of the robot.

To demonstrate the crawling capability of the OVFSCR on different substrate surfaces comprising slippery, dry smooth, and semi-rough surfaces, we carried out three sets of experiments under the same vacuum pressure of 99.8 kPa and the same duration (i.e., *t*_on_ = 0.8 s, *t*_off_ = 0.7 s). The OVFSCR’s locomotion experiments were recorded by a high-resolution camera at 50 frames per second, the recorded video frames were processed by imaging processing software, and some snapshots of its crawling motion on flat surfaces were then extracted at different moments. The final results are shown in Figure 4 and Supplementary Movie. Overall, these measurement results indicate that the robot can move forward continuously and have distinctly different crawling speeds on diverse substrate surfaces. The crawling speed is defined as the ratio of locomotion distance to time.

**Figure 5:**
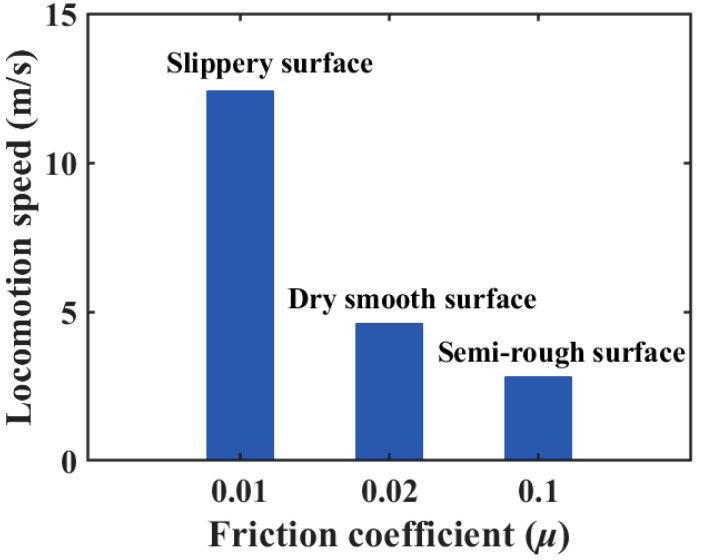
Locomotion speed versus friction coefficient for three different types of substrate surfaces.

From the experimental results in Figure 4, we can see that the locomotion speed (12.4 mm/s) on the slippery surface is fastest, and the speed (4.6 mm/s) on the dry smooth surface is quicker than that (2.8 mm/s) on the semi-rough surface, which is caused by different friction coefficients. It is demonstrated from the bar graph (Figure 5) that different substrate materials have different friction coefficients that induce different frictional forces between the robot and contact surfaces, thereby affecting the crawling locomotion speed. It follows that the locomotion speed decreases with the increase in the friction coefficient within a certain range of friction coefficients. The smaller the friction coefficient, the smaller the frictional resistance, and the faster the locomotion speed. The robot is able to operate under some harsh conditions such as slippery or lubricated surfaces, demonstrating its adaptability to unstructured environments.

As compared to undulatory, walking, or crawling gaits of other positive-pressure actuated SCRs which require multiple relatively complex actuation sequences [24,31,37], the OVFSCR is characterized by fast contractive and recovery capability, and an intermittent crawling gait that only requires two repeated actuation sequences (i.e., contracting and recovering), which is the main reason for fulfilling rapid locomotion.

### 3.2. Pushing Objects and Passing through Tubes

In addition to locomoting on flat surfaces as described above, the OVFSCR is capable of walking and turning on non-planar surfaces, allowing it to navigate into constrained spaces and perform various tasks.

To characterize the locomotion performance and manipulation capacity of the OVFSCR in a confined tube, we conducted four different groups of experiments under the same conditions. Similar to the above planar crawling experiments, we can also extract several snapshots of the robot inside round tubes at various moments.

**Figure 6:**
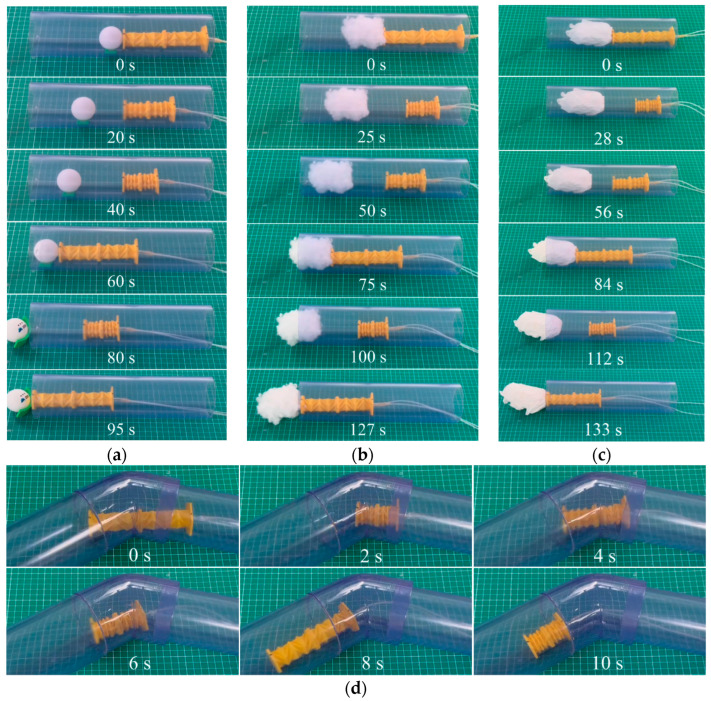
Demonstrations of the OVFSCR crawling inside round tubes. The robot (**a**) pushes a small ping-pong ball forward; (**b**) cleans a wad of cotton; (**c**) propels a lump of paper; and (**d**) passes through a corner of 135° by self-navigation along a predetermined direction.

It can be obviously observed from Figure 6 and Supplementary Movie that the robot has multiple functions, such as pushing or removing target objects, crawling, and turning.

Potential uses of the OVFSCR include cleaning blockages inside tubes, monitoring the interior of pipelines, channels, or groups of buildings, and conducting search tasks. Figure 6a–c and Supplementary Movie exhibit that the robot pushes a small ping-pong ball, a lump of paper, and a wad of cotton out of a transparent tube to demonstrate its transporting or cleaning capability, which is mainly attributed to the contribution of the large thrust generated by the vacuum pressure and the rapid deformation performance. The soft robot can also carry a camera to visually inspect internal defects in tubes, clear away small barriers, pass through dense smoke, and perform transportation missions and other tasks.

Additionally, the OVFSCR also has turning capability. Figure 6d and Supplementary Movie exhibit the turning motion by self-navigation, indicating that the robot can navigate itself when turning a corner or faced with only one alternative path. It can passively adapt to narrow spaces to move along a predetermined direction. This performance is superior to that of some other SCRs [10,12,15,24,38,39,40] whose turning angles are detrimentally limited by slippage and affected by the tardy response time of DE, positive-pressure, or SMA actuation modes, resulting in slow turning speed of those SCRs. By contrast, the OVFSCR exhibits a relatively fast linear speed of 11.9 mm/s and turning speed of 13.5°/s; this performance is better than that of those SCRs, even including a few vacuum-driven soft robots (Table 1). This is due to the combination of the vacuum-actuated mode and origami actuator design, which enable it to contract and recover in a short time, and thus it can rapidly turn and adapt to limited narrow spaces.

### 3.3. Climbing Slopes

**Figure 7:**
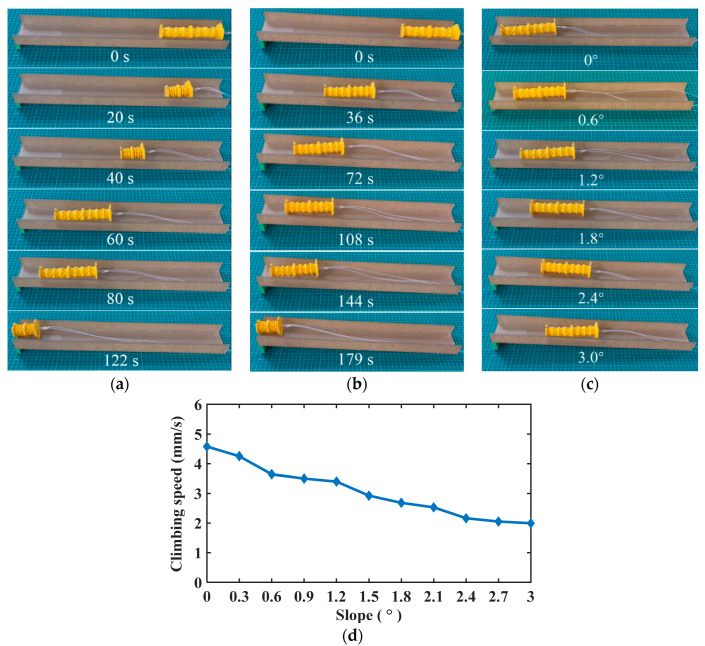
Demonstrations of the OVFSCR climbing slopes. The robot climbs (**a**) a slope of 1.5°; (**b**) a slope of 3° at different moments; and (**c**) different slopes at the same moment *t* = 60 s. (**d**) The climbing speed versus the slope.

The OVFSCR can also climb a slope. The vacuum pressure provides power for actuating the robot, and the inclined surface of the rear foot comes into surface contact with the slope to prevent backward slippage when the vacuum is released, which is a critical factor to climbing slopes. Since the OVFSCR contracts toward the center upon exerting a vacuum, its barycenter is basically close to the slope, which lowers the probability of retreating during climbing. Under the same vacuum pressure of 99.8 kPa and the same duration (i.e., *t*_on_ = 0.8 s, *t*_off_ = 0.7 s), we conducted climbing experiments on a dry smooth surface with a friction coefficient of 0.02 to explore the relation between the climbing speed and the slope. It is clearly revealed from Figure 7a–d that the climbing speed decreases with the increase in the slope.

Figure 7a–c show the climbing progression of the OVFSCR on different slopes. The robot has a climbing speed of 2.9 mm/s on a slope of 1.5°, which is quicker than that on a slope of 3° (i.e., 2.0 mm/s); various videos of climbing different slopes are available in Supplementary Movie. However, the climbing capability of our robot still falls behind some SCRs climbing vertical wall surfaces [31,32,35,36]. Therefore, the future research goal is to elevate the climbing performance and adapt to different terrains by adding vacuum suckers and optimizing the control strategy.

### 3.4. Carrying Different Payloads

In some applications involving field exploration, search and rescue, and camouflage reconnaissance, soft robots are required to carry some devices and bear weights, and thus it is a challenging task for the OVFSCR to carry payloads. Under the same experimental conditions as in Section 3.3, we executed load-bearing experiments to investigate its load-carrying capability. Different weights were attached to the robot, and the average locomotion speed was also measured and recorded.

**Figure 8:**
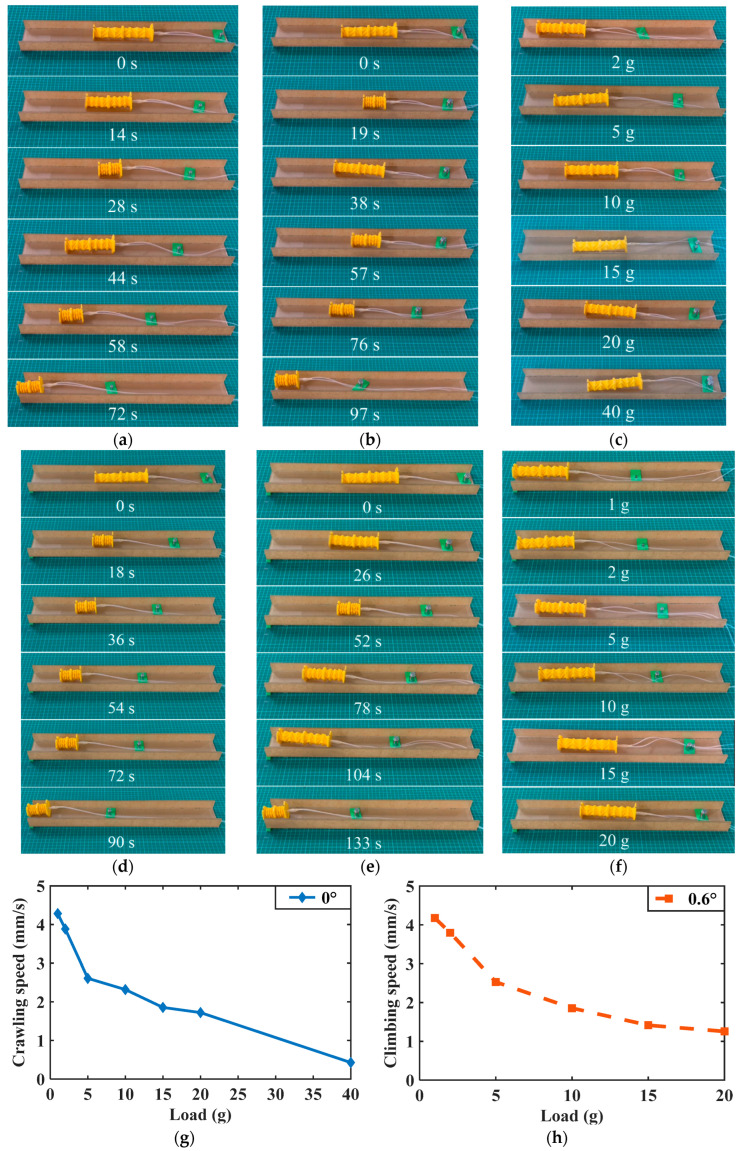
Demonstrations of the OVFSCR carrying payloads. The robot carries (**a**) a payload of 10 g at an average speed of 2.3 mm/s; (**b**) a payload of 20 g at an average speed of 1.7 mm/s on a flat surface; (**c**) different payloads on the flat surface at same moment *t* = 30 s; (**d**) a payload of 10 g at an average speed of 1.9 mm/s on a slope of 0.6°; (**e**) a payload of 20 g at an average speed of 1.3 mm/s on a slope of 0.6°; and (**f**) different payloads on the slope of 0.6° at same moment *t* = 35 s. (**g**) The crawling speed versus the payload on the flat dry smooth surface. (**h**) The climbing speed versus the payload on the inclined dry smooth surface with a slope of 0.6°.

Figure 8a,b exhibit the climbing motion of the OVFSCR carrying payloads of 10 g and 20 g at different moments. Figure 8c show the climbing motion of the robot carrying different payloads on the flat dry smooth surface at the same moment *t* = 30 s. As expected, the crawling speed decreases with the enhancement of payloads (Figure 8g), and the increased payload restricts contractile deformation of the robot’s main body.

Different videos of the robot carrying different payloads on the flat surface are available in Supplementary Movie. We find that the robot’s bearing capability is largely affected by tethered tubes, which requires the development of untethered autonomous SCRs in the future. In addition to studying payloads carried by the OVFSCR on the flat surface, we also conducted several experiments to examine its bearing capability on slopes. Notably, the robot can even climb a slope of 0.6° while taking a certain payload (see Figure 8d,f,h, Supplementary Movie).

It can be observed from Figure 8h that the climbing speed decreases with the increase in the payload. Despite this, the soft robot can successfully climb a slope of 0.6° while carrying a payload of 20 g. However, our robot’s bearing capability and locomotion speed on the slope are still lower than that of some SCRs [24,27,31,36,40]. The main reason for this is that our robot lacks sufficient static frictional or adhesion forces to gain a firm grip and prevent slipping. In light of this, incorporating adhesion structures (e.g., adding sucker or gecko adhesion feet) to the OVFSCR will be one of our future research directions, as it can both improve its carrying-load capacity and facilitate vertical climbing ability.

To reflect our robot’s superiority, we compared it with other SCRs in terms of other performance metrics including self-weight, payload/self-weight, response time in one cycle, stability, and durability (Table 2). As compared to some SCRs [15,24,41] based on Pos-pressure, SMA, and DE, the OVFSCR has relatively light self-weight, high payload/self-weight, quicker response time, better stability, and more reliable durability, which benefits from the unique combination of mirrored origami actuators and the vacuum actuation method.

### 3.5. Travelling across Different Media and Bearing Impact

**Figure 9:**
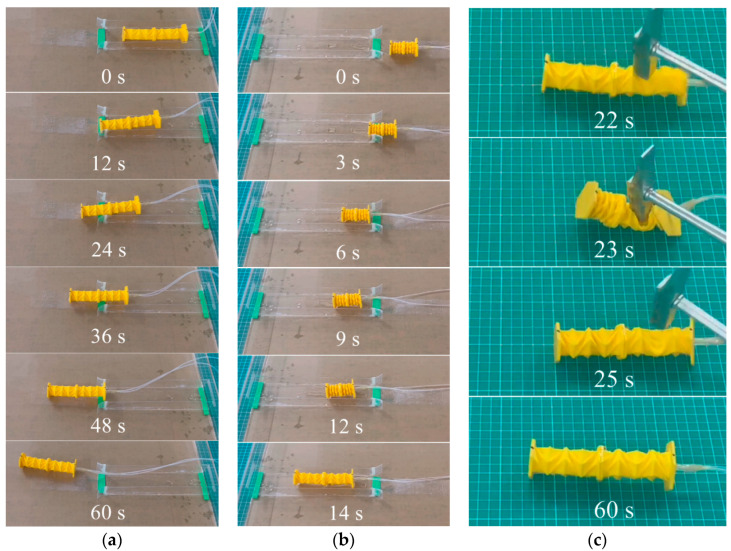
Demonstrations of the OVFSCR travelling across different media. The robot (**a**) walks from an oblique smooth acrylic plate in air to a sink filled with a certain amount of water; (**b**) travels from a sink filled with water to an oblique acrylic plate in air; and (**c**) suffers from a vertical impact load and becomes flattened; it can quickly recover its original shape and locomote when the load is withdrawn.

Lastly, we demonstrated the potential applications of the OVFSCR traversing different media, which showed it can walk from a slope in the air to water without impairing its crawling performance, and vice versa (see Figure 9a,b, Supplementary Movie). This provides a new way of thinking for real-time online monitoring and detection in harsh environments. Owing to the unique advantage of combining foldable origami actuators with the vacuum-actuated mode, our robot can operate and walk with ease in vacuum, watery, and even amphibious environments, whereas it may be an austere challenge for some SCRs driven by positive pressure and SMA.

Furthermore, a vertical impact load was imposed on the OVFSCR during the crawling motion to test its ability to withstand strong impacts (Figure 9c, Supplementary Movie). We can use the same vacuum pressure of 99.8 kPa to actuate the robot and observe the deformed shape after applying an impact load. The robot can bear large forces up to 10.88 N during the operation without being damaged, which may be the result of the completely soft feature of the TPE material and the quickly restorable deformation capability of the foldable origami actuator. Accounting for the cross-section (a square with a side length of 15 mm) of a vertical load of 1088 g, the pressure (0.048 MPa) generated is comparable to that induced by a person (weighing 193 kg with a contact area of 250 × 80 × 2 mm^2^), indicating that the robot can withstand the strong impact load without suffering from mechanical damage. It is expected that our robot can accomplish some arduous tasks under severe conditions such as large impacts. However, this is a challenging task for several SCRs containing rigid components or even some positive-pressure powered SCRs.

## 4. Conclusions and Future Work

To summarize, we proposed a biomimetic OVFSCR with a Kresling crease pattern, analogous to a soft worm, which integrates asymmetrical foldable origami actuators with a vacuum-actuated mode to enable controllable unidirectional locomotion, and an efficient dynamic model was developed to describe the robot’s locomotion capability. This OVFSCR features the abilities of an easy control method, crawling on diverse substrate surfaces, removing obstacles, adaptively navigating, climbing different slopes, carrying weight, multimodal locomotion, travelling across different media, and bearing a large load.

One benefit of the OVFSCR is that the vacuum-actuated origami actuator empowers the robot to achieve periodic continuous forward crawling motion with one contraction and one restoration per cycle, which is different from other SCRs that need constant actuation. The capability of the robot to fulfill high motion efficiency depends on not only the applied vacuum pressure, but also the asymmetric structural design of low front and high rear hexahedral feet, creating a biased frictional force between two feet to enable locomotion on various contact surfaces. Under the same actuation sequences and the vacuum pressure of 99.8 kPa, our robot exhibits a crawling speed of 12.3 mm/s on a slippery surface, a climbing speed of 2.0 mm/s on a slope of 3°, and a turning speed of 13.5° inside a curved circular tube, and it can carry a certain payload, withstand impact, and possess multifunctional characteristics, thereby enriching the library of existing SCRs. Furthermore, by combining vacuum actuation with an asymmetrical structural design to actively control the contact interaction of the OVFSCR’s rear foot with the ground, the robot realizes successive forward crawling on different contact surfaces, which is often difficult for other types of SCRs with a passive control mechanism (e.g., adding extra anchored feet or sucker structures).

Although self-powered autonomous untethered soft robots are more popular in practical engineering application fields, the previously proposed soft crawling, climbing, grasping, swimming, and growing robots are mostly tethered. Presently, it remains a demanding challenge to develop autonomous, controllable, untethered SCRs. In the future, we will be committed to improving movement efficiency, developing autonomous power systems, and optimizing control strategies to enable foldable and restorable SCRs for wide use in various terrains covering topographic prospecting, camouflage information reconnaissance, medical diagnosis, unknown territory exploration, and underwater pipeline inspection. Furthermore, we will devise and implant microsensors, microcontrollers, micro-compressors, microvalves, and microtubes into SCRs for autonomously untethered control and manipulation.

## Figures and Tables

**Table 1:** A comparison of average linear speed and turning speed of different SCRs.

SCRs	Actuation Method	Tethered	BL (mm)	Linear Speed (mm/s)	Turning Speed (°/s)
Our robot	Vacuum	Yes	143	11.9	13.5
Robot [32]	Vacuum	Yes	135	5.0	3.5
Robot [35]	Vacuum	Yes	135	10.3	15.09
Robot [12]	Pos-pressure	Yes	135.7	7.2	Unknown
Robot [24]	Pos-pressure	No	650	5.0	~0.21
Robot [38]	Pos-pressure	Yes	154	5.1	1.63
Robot [15]	SMA	Yes	196	3.6	0.15
Robot [39]	SMA	Yes	200	5.25	~0.35
Robot [10]	DE	Yes	170	5.3	Unknown
Robot [40]	DE	No	110	4.16	~0.3

Pos-pressure, positive-pressure; SMA, shape memory alloy; DE, dielectric elastomer; BL, body length.

**Table 2:** A comparison of some performance metrics of diverse SCRs.

SCRs	Actuation Method	Self-Weight (g)	Payload/Self-Weight	Response Time (s)	Stability	Durability
Our robot	Vacuum	22.5	~178%	~1.5	Yes	Yes
Robot [24]	Pos-pressure	3800	~58%	~13.0	Yes	No
Robot [15]	SMA	63.0	Unknown	~15.0	No	No
Robot [41]	DE	5.0	~200%	~3.8	No	No

Pos-pressure, positive-pressure; SMA, shape memory alloy; DE, dielectric elastomer.

## Data Availability

Data are contained within the article.

## Supplementary Materials

The following supporting information can be downloaded at: https://www.mdpi.com/article/10.3390/biomimetics9090541/s1, Supplementary Movie.

## References

[1] Wu Y., Yim J.K., Liang J., Shao Z., Qi M., Zhong J., Luo Z., Yan X., Zhang M., Wang X. (2019). Insect-scale fast moving and ultrarobust soft robot. Sci. Robot..

[2] Gu G., Zou J., Zhao R., Zhao X., Zhu X. (2018). Soft wall-climbing robots. Sci. Robot..

[3] Zhang Z., Long Y., Chen G., Wu Q., Wang H., Jiang H. (2023). Soft and lightweight fabric enables powerful and high-range pneumatic actuation. Sci. Adv..

[4] Li G., Chen X., Zhou F., Liang Y., Xiao Y., Cao X., Zhang Z., Zhang M., Wu B., Yin S. (2021). Self-powered soft robot in the Mariana Trench. Nature.

[5] Greer J.D., Blumenschein L.H., Alterovitz R., Hawkes E.W., Okamura A.M. (2020). Robust navigation of a soft growing robot by exploiting contact with the environment. Int. J. Robot. Res..

[6] Li W.-B., Zhang W.-M., Zou H.-X., Peng Z.-K., Meng G. (2018). Multisegment annular dielectric elastomer actuators for soft robots. Smart. Mater. Struct..

[7] Chen Y., Zhao H., Mao J., Chirarattananon P., Helbling E.F., Hyun N.P., Clarke D.R., Wood R.J. (2019). Controlled flight of a microrobot powered by soft artificial muscles. Nature.

[8] Galloway K.C., Becker K.P., Phillips B., Kirby J., Licht S., Tchernov D., Wood R.J., Gruber D.F. (2016). Soft robotic grippers for biological sampling on deep reefs. Soft Robot..

[9] Wehner M., Truby R.L., Fitzgerald D.J., Mosadegh B., Whitesides G.M., Lewis J.A., Wood R.J. (2016). An integrated design and fabrication strategy for entirely soft, autonomous robots. Nature.

[10] Xu L., Chen H.-Q., Zou J., Dong W.-T., Gu G.-Y., Zhu L.-M., Zhu X.-Y. (2017). Bio-inspired annelid robot: A dielectric elastomer actuated soft robot. Bioinspir. Biomim..

[11] Xiao Y., Mao J., Shan Y., Yang T., Chen Z., Zhou F., He J., Shen Y., Zhao J., Li T. (2020). Anisotropic electroactive elastomer for highly maneuverable soft robotics. Nanoscale.

[12] Shepherd R.F., Ilievski F., Choi W., Morin S.A., Stokes A.A., Mazzeo A.D., Chen X., Wang M., Whitesides G.M. (2011). Multigait soft robot. Proc. Natl. Acad. Sci. USA.

[13] Fang H., Zhang Y., Wang K.W. (2017). Origami-based earthworm-like locomotion robots. Bioinspir. Biomim..

[14] Tang Z., Lu J., Wang Z., Chen W., Feng H. (2020). Design of a new air pressure perception multi-cavity pneumatic-driven earthworm-like soft robot. Auton. Robot..

[15] Wang W., Lee J.-Y., Rodrigue H., Song S.-H., Chu W.-S., Ahn S.-H. (2014). Locomotion of inchworm-inspired robot made of smart soft composite (SSC). Bioinspir. Biomim..

[16] Wang J., Min J., Fei Y., Pang W. (2019). Study on nonlinear crawling locomotion of modular differential drive soft robot. Nonlinear Dynam..

[17] Sun L., Chen Z., Bian F., Zhao Y. (2019). Bioinspired soft robotic caterpillar with cardiomyocyte drivers. Adv. Funct. Mater..

[18] Jin G., Sun Y., Geng J., Yuan X., Chen T., Liu H., Wang F., Sun L. (2021). Bioinspired soft caterpillar robot with ultra-stretchable bionic sensors based on functional liquid metal. Nano Energy.

[19] Cortez R., Sandoval-Chileño M.A., Lozada-Castillo N., Luviano-Juárez A. (2024). Snake robot with motion based on shape memory alloy spring-shaped actuators. Biomimetics.

[20] Luo M., Agheli M., Onal C.D. (2014). Theoretical modeling and experimental analysis of a pressure-operated soft robotic snake. Soft Robot..

[21] Jayaram K., Full R.J. (2016). Cockroaches traverse crevices, crawl rapidly in confined spaces, and inspire a soft, legged robot. Proc. Natl. Acad. Sci. USA.

[22] Liu R., Zheng H., Hliboký M., Endo H., Zhang S., Baba Y., Sawada H. (2024). Anatomically-inspired robotic finger with SMA tendon actuation for enhanced biomimetic functionality. Biomimetics.

[23] Sun Y., Abudula A., Yang H., Chiang S.-S., Wan Z., Ozel S., Hall R., Skorina E., Luo M., Onal C.D. (2021). Soft mobile robots: A review of soft robotic locomotion modes. Curr. Robot. Rep..

[24] Tolley M.T., Shepherd R.F., Mosadegh B., Galloway K.C., Wehner M., Karpelson M., Wood R.J., Whitesides G.M. (2014). A resilient, untethered soft robot. Soft Robot..

[25] Rafsanjani A., Zhang Y., Liu B., Rubinstein S.M., Bertoldi K. (2018). Kirigami skins make a simple soft actuator crawl. Sci. Robot..

[26] Calderón A.A., Ugalde J.C., Chang L., Zagal J.C., Pérez-Arancibia N.O. (2019). An earthworm-inspired soft robot with perceptive artificial skin. Bioinspir. Biomim..

[27] Liao B., Zang H., Chen M., Wang Y., Lang X., Zhu N., Yang Z., Yi Y. (2020). Soft rod-climbing robot inspired by winding locomotion of snake. Soft Robot..

[28] Chen Y., Hu B., Zou J., Zhang W., Wang D., Jin G. (2020). Design and fabrication of a multi-motion mode soft crawling robot. J Bionic. Eng..

[29] Wang N., Chen B., Ge X., Zhang X., Wang W. (2021). Modular crawling robots using soft pneumatic actuators. Front. Mech. Eng..

[30] Chen S., Cao Y., Sarparast M., Yuan H., Dong L., Tan X., Cao C. (2019). Soft crawling robots: Design, actuation, and locomotion. Adv. Mater. Technol..

[31] Verma M.S., Ainla A., Yang D., Harburg D., Whitesides G.M. (2018). A soft tube-climbing robot. Soft Robot..

[32] Robertson M.A., Paik J. (2017). New soft robots really suck: Vacuum-powered systems empower diverse capabilities. Sci. Robot..

[33] Ainla A., Verma M.S., Yang D., Whitesides G.M. (2017). Soft, rotating pneumatic actuator. Soft Robot..

[34] Jiao Z., Zhang C., Wang W., Pan M., Yang H., Zou J. (2019). Advanced artificial muscle for flexible material-based reconfigurable soft robots. Adv. Sci..

[35] Qin L., Liang X., Huang H., Chui C.K., Yeow R.C.-H., Zhu J. (2019). A versatile soft crawling robot with rapid locomotion. Soft Robot..

[36] Li D., Fan D., Zhu R., Lei Q., Liao Y., Yang X., Pan Y., Wang Z., Wu Y., Liu S. (2023). Origami-inspired soft twisting actuator. Soft Robot..

[37] Tang Y., Zhang Q., Lin G., Yin J. (2018). Switchable adhesion actuator for amphibious climbing soft robot. Soft Robot..

[38] Zou J., Lin Y., Ji C., Yang H. (2018). A reconfigurable omnidirectional soft robot based on caterpillar locomotion. Soft Robot..

[39] Seok S., Onal C.D., Cho K.-J., Wood R.J., Rus D., Kim S. (2013). Meshworm: A peristaltic soft robot with antagonistic nickel titanium coil actuators. IEEE-ASME T. Mech..

[40] Cao J., Qin L., Liu J., Ren Q., Foo C.C., Wang H., Lee H.P., Zhu J. (2018). Untethered soft robot capable of stable locomotion using soft electrostatic actuators. Extreme Mech. Lett..

[41] Li T., Zou Z., Mao G., Yang X., Liang Y., Li C., Qu S., Suo Z., Yang W. (2019). Agile and resilient insect-scale robot. Soft Robot..

